# A Chinese pedigree with Brown-Vialetto-Van Laere syndrome due to two novel mutations of SLC52A2 gene: clinical course and response to riboflavin

**DOI:** 10.1186/s12881-019-0811-1

**Published:** 2019-05-07

**Authors:** Kaili Shi, Zhen Shi, Huifang Yan, Xiaodong Wang, Yanling Yang, Hui Xiong, Qiang Gu, Ye Wu, Yuwu Jiang, Jingmin Wang

**Affiliations:** 1 Department of Pediatrics, Peking University First Hospital, Beijing, 100034 China; 2 Department of Neurology, Children’s Hospital of Shanxi, Taiyuan, 030013 China; 3 Beijing Key Laboratory of Molecular Diagnosis and Study on Pediatric Genetic Diseases, Peking University First Hospital, Beijing, 100034 China; 4 Cipher Gene, LCC, Beijing, 100080 China; 5 Key Laboratory for Neuroscience, Ministry of Education/National Health and Family Planning Commission, Peking University, Beijing, 100034 China

**Keywords:** Brown-Vialetto-Van Laere syndrome, *SLC52A2*, Sensorineural hearing loss, Breath holding spells

## Abstract

**Background:**

Brown-Vialetto-Van Laere Syndrome (BVVLS), a rare neurological disorder characterized by motor, sensory, and cranial neuronopathies, is mainly associated with defective riboflavin transporters encoded by SLC52A2 and SLC52A3 genes. Clinical outcomes have been shown to be improved significantly by high-dose riboflavin supplementation. The aim of this study was to identify genetic causes and further evaluate the clinical course and response to riboflavin in a Chinese pedigree with BVVLS.

**Case presentation:**

We report the novel compound heterozygous variants c.1328G>A p.(Cys443Tyr) and c.1022_1023insC p. (Leu341Profs*103) of SLC52A2 gene in a female proband who presented in our out-patient clinic at the age of one-year-old with progressive mental and motor regression, breath holding, and brain stem dysfunction including facial weakness, hearing loss, dysphagia. Following high-dose riboflavin supplementation, the respiratory insufficiency and mental, motor, and bulbar function improved. However, sensorineural hearing loss was not improved. The missense variant site was highly conserved. Both variants were not found in the population database gnomAD. The two variants were inherited from her mother and father, respectively. Both variants were predicted to be deleterious by Polyphen2, Mutation taster, and SIFT and were classified as likely pathogenic according to the ACMG guideline.

**Conclusions:**

Two novel pathogenic variations of SLC52A2 gene were firstly found from a Chinese pedigree with BVVLS. Clinical outcomes could be improved by early diagnosis and riboflavin supplementation.

## Background

Brown-Vialetto-Van Laere Syndrome (BVVLS) was first described by Brown in 1894 [1] and later by Vialetto [2] and Van Laere [3]. BVVLS is a rare autosomal recessive neurological disorder characterized by axial and appendicular weakness, sensory neuronopathy, gait ataxia, bulbar palsy, sensorineural hearing loss, optic atrophy, and facial weakness. Variants in riboflavin transporter genes *SLC52A2* (coding for RFVT2) and *SLC52A3* (coding for RFVT3) were demonstrated to be the disease-causing genes in this neurodegenerative disorder, which was formerly known as BVVL syndrome [4, 5] and is renamed riboflavin transporter deficiency [6]. The identification of clinical variants in RFVT genes has led to a better understanding of the pathophysiology of associated neurological disorders and guided disease management in affected patients [7]. A recent study reported 37 patients with molecular diagnosis of BVVLS caused by variants in the SLC52A2 gene [6]. High-dose riboflavin supplementation has shown to significantly improve clinical outcomes, particularly if initiated soon after the onset of symptoms [8]. Thus far, only one clinically diagnosed BVVLS case with severe sleep-disordered breathing was reported among Chinese populations [9]. In this report, the authors describe the clinical course and responsiveness to riboflavin of a Chinese BVVLS patient carrying two novel pathogenic variants of *SLC52A2*.

## Case presentation

A one-year-nine-month old Chinese girl with symptoms of hearing loss and retrogression of speech and movement since one-year-old presented in our out-patient service. The patient was responsible to teasing, and her neck stood firmly at the age of 5 months. She was able to flip over her body at the age of 8 months, responded when her name was called, and was able to call mom and dad at the age of 9 months. However, her motor development lagged behind her peers obviously. She was not able to sit and crawl independently at one-year-old. Since then, the patient gradually lost her response to surroundings and had lack of facial expression and hypotonia, especially weakness in upper limbs, including loss of hand agility and lack of grabbing. Other symptoms included choking when drinking and swallowing difficulty, but seizure was not observed. Her body weight decreased from 10.5 kg to 8 kg after the symptoms manifested. She started holding her breath for 1–2 min frequently after crying, starting at the age of 10 months. Cyanotic breath holding spells (BHS) occurred on an average of 10 times/day. She was the first child of non-consanguineous parents. The first and second pregnancies were ceased by her parents, and the 3rd pregnancy was aborted because the embryo stopped developing. She was born at 32^+ 6^ weeks of gestation through cesarean section because her mother suffered from pregnancy-induced hypertension. Her birth weight was 1.36 kg, and her newborn hearing screening result was unremarkable. When she came to the clinic at one-year-nine-month, her height was 77 cm, her weight was 8 kg, and her head circumference was 45 cm, all lagging behind children of the same age. She could only control her head, and she showed poor visual fixation and sound tracking. Physical examination showed generalized weakness, especially the upper extremities, hypotonia of limbs, weak gag reflex, absent of patellar tendon reflex, and negative bilateral Babinski sign. Ammonia, serum lactic acid, hepatorenal function, microelements, and serum amino acids were normal. Acylcarnitine profile showed mild abnormalities including mild elevation of octanoyl carnitine (C8): 0.33 μmol/L (0.01–0.30 μmol/L) and decanoyl carnitine (C10): 0.50 μmol/L (0.01–0.35 μmol/L). Other acylcarnitine species were within the normal ranges. Her urine organic acid analysis showed mild elevated pyruvic acid and lactic acid.

Magnetic resonance imaging (MRI) of the brain showed no contrast and visual-evoked response. Video electroencephalogram and echocardiography were normal. Electromyogram was neurogenic with fibrillation activity. Nerve conduction studies showed denervation without sensory response of the sural and median nerves but normal motor velocities. Brainstem auditory-evoked responses revealed severe sensorineural hearing loss. The ophthalmologic examination was normal. With the consent of the child’s parents, genetic testing was performed. No variant was found in the SMN (survival motor neuron) gene, which encodes survival motor neuron protein. Her karyotype analysis was normal.

## Materials and methods

### Patient

Clinical data of the family were collected and analyzed. Follow-up information was obtained by telephone. The study was approved by the Ethics Committee of Peking University First Hospital. Informed consent was obtained from the parents of this proband.

### Genetic analysis

Peripheral venous blood was collected from the proband and her parents. DNA extraction was performed as previously described [10]. Whole-exome sequencing was carried out. The sequencing libraries were prepared, and the xGen Exome Research Panel probes (IDT, USA) were used to enrich the target sequences. The enriched DNA was sequenced by Novoseq 6000 (Illumina, USA). Sequence variants were checked with population databases gnomAD (http://gnomad-old.broadinstitute.org/). Pathogenicity was predicted by Polyphen2, Mutation Taster, and SIFT. Variant pathogenicity was interpreted according to the American College of Medical Genetics (ACMG) guidelines [11]. The variants were further confirmed by Sanger sequencing.

## Results

**Fig. 1:**
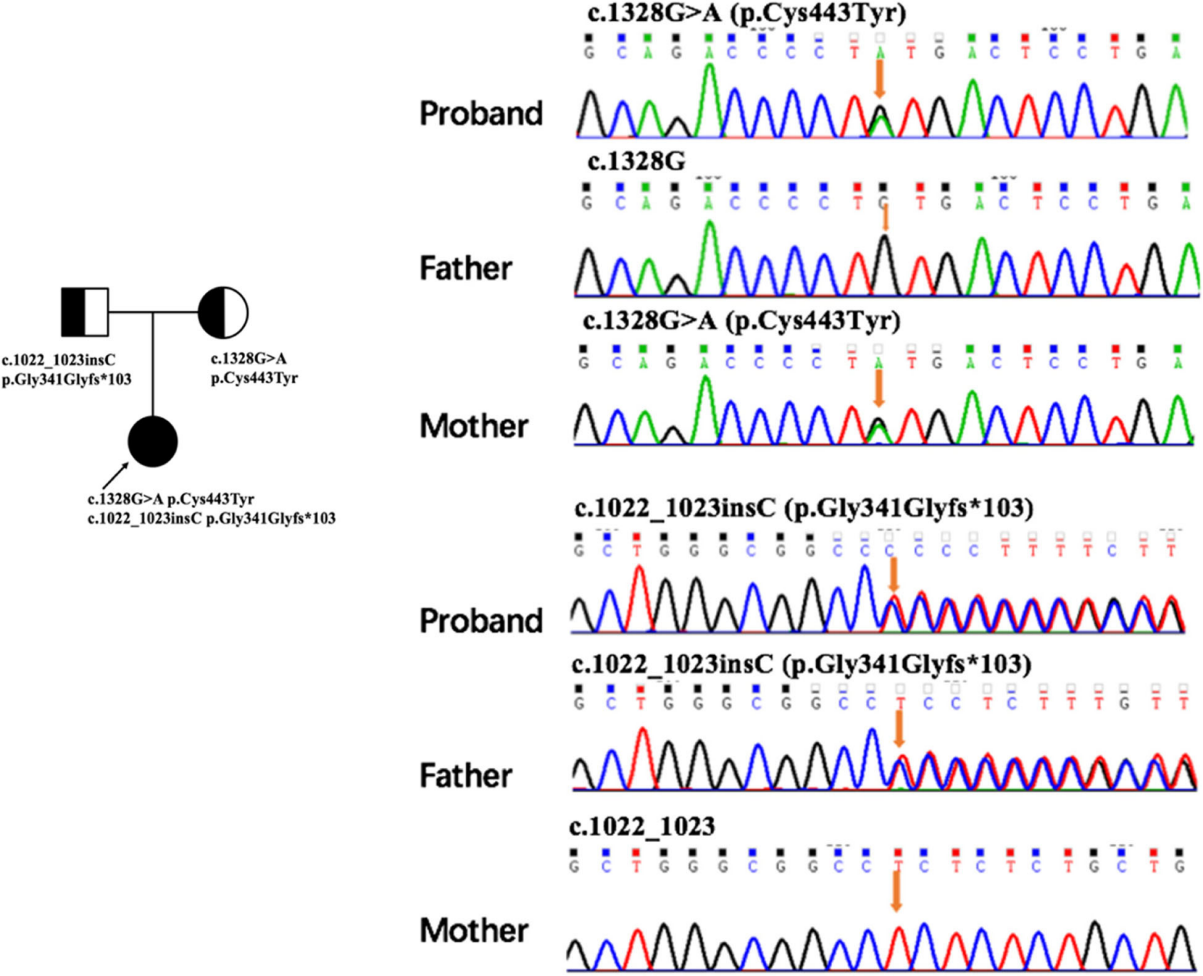
The genogram of this family and the variants of *SLC52A2* c.1328G>A, p.Cys443Tyr, and c.1022_1023insC, Leu341Profs*103 in BVVLS proband and parents

Two novel variants in SLC52A2 gene were identified: a 1022 C insertion resulting in a frame-shift protein stopping at 103th amino acid and a 1328G-A transition variant resulting in a 443Cys-to-Tyr substitution. The Sanger sequencing confirmed the compound heterozygous state of the variants in the affected individual with the mother being the heterozygous carrier of the c.1328G>A, p.Cys443Tyr and the father being the carrier of the c.1022_1023insC, Leu341Profs*103 variant (Fig. 1). The variant c.1328G>A, p.Cys443Tyr was predicted to be deleterious by Polyphen2, Mutation taster, and SIFT. Both variants were absent in the population database gnomAD and were classified as likely pathogenic by the ACMG guideline.

The patient was diagnosed with BVVLS and prescribed to start on oral riboflavin supplementation at a dose of 15 mg/kg/d first and increased to 50 mg/kg/d gradually. The patient showed no adverse effects for 10 months. Her muscle weakness improved as she could turn over now, held a feeding bottle by herself, and choked less frequently. The BHS also improved significantly, from about 10 times a day previously to 2–3 times now. However, her hearing loss remained unchanged, and artificial cochlea implantation is on the plan.

## Discussion and conclusions

BVVLS is a rare neurological disease with progressive pontobulbar palsy, sensorineural hearing loss, and respiratory compromise and is caused by compound heterozygous or homozygous variants in the SLC52A3 and SLC52A2 genes [4, 5]. The patient described here displayed bulbar palsies, sensorineural hearing loss, and progressive axonal sensorimotor neuropathy (general weakness especially for the upper extremities) [6]. In addition, she had BHS, which was rarely reported in patients with BVVLS, except in a patient with riboflavin transporter deficiency and SLC52A2 variant (c.917G>A; p. Gly306Glu); this patient had remarkable breath-holding spells since 6 months, but the evolution of the symptom and the relationship to the disease was not analyzed [12]. Our patient did not have anemia. Her cyanotic BHS appeared along with other symptoms of BVVLS; such symptoms were significantly improved by the riboflavin treatment. A recent case control study reported maturation delay in myelination of the brainstem as assessed from the inter-peak latencies on brainstem auditory. The observation of BHS in our patient is consistent with the study that suggests that the neurodegeneration of brainstem myelination might be the potential cause of BHS [13].

**Fig. 2:**
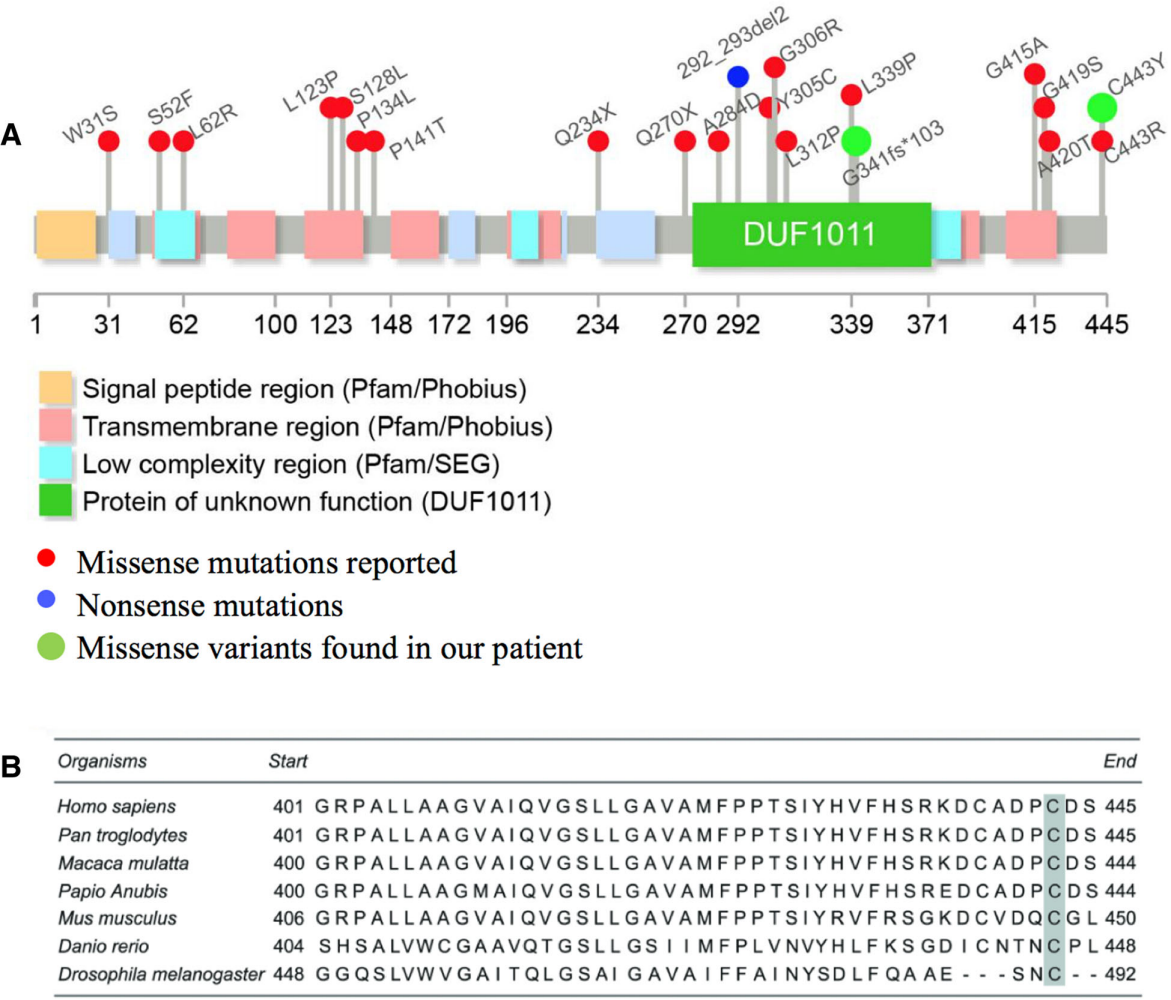
**a** Functional domains and regions in SLC52A2 gene as indicated. Red dots: missense mutations; Blue dots: nonsense mutations; Green dots: missense variants found in our patient. **b** Evolutionary conservation of cysteine residue at position 443 (shade in grey) in the SLC52A2 gene among species

SLC52A2 gene encodes for a transmembrane protein (hRFVT2) containing 445 amino acids and 11 transmembrane helices. hRFVT2 mediates the cellular uptake of riboflavin. The water-soluble vitamin riboflavin is converted into the coenzymes flavin mononucleotide (FMN) and flavin adenine dinucleotide (FAD), which are essential for normal cellular functions. These coenzymes are indispensable in a number of cellular processes, such as normal mitochondrial function [14]. hRFVT2 is expressed primarily in the brain. Transportation of riboflavin into the brain is impaired and abnormal metabolism results in hRFVT2 deficiency [15]. Pathogenic variants of SLC52A2 gene are distributed throughout the gene in regions encoding transmembrane, intracellular, and extracellular loops [8, 16]. Only nonsynonymous pathogenic variants in the homozygous or compound heterozygous state have been reported to date [17, 18]. The two novel nonsynonymous variants found in our patient are located in the protein functional domain and extracellular loop region (Fig. 2a). Although variant 443Cys-to-Tyr is located close to the end of coding protein, it is in an important loop area. The missense variant (c.1328G>A, p. Cys443Tyr) identified in our patient is predicted to be functionally damaging by several bioinformatics software. Cysteine at position 443 is evolutionarily conserved among multiple species (Fig. 2b). A variant in the same amino acid of 443Cys-to-Arg was reported to be pathogenic in the study of Gahl et al. [19]. Limited information is known about the function of this region. Further studies are necessary to reveal the pathogenic mechanism of this presumably important residue.

Although infants with early-onset BVVL syndrome rapidly become ventilator-dependent and usually die in the first year of life, treatment with riboflavin can partially ameliorate the progression of the disease [8] and decrease the mortality rate [5, 6]. A recent review demonstrated that all patients who were not treated with riboflavin showed gradual deterioration. The review also showed that among 20 RFVT2 deficient patients, 12 patients (60%) suffering from neurological symptoms had improved and eight patients (40%) remained stable after initiation of riboflavin [6]. Our patient exhibited continue improvement with dosage increments in terms of normal muscle tone, bulbar function, and BHS.

In this case report, we identified two novel variants from a Chinese BVVLS patient through genetic testing. The results show the importance of early diagnosis for symptom management, which can lead to improvement in clinical outcomes in patients with the disease. Physicians should consider this rare neurologic disease when evaluating patients with similar symptoms. Genetic testing may help identify and confirm the BVVL syndrome.

## Abbreviations

ACMG: American College of Medical Genetics and Genomics
BHS: Breath holding spells
BVVLS: Brown-Vialetto-Van Laere syndrome
FAD: Flavin adenine dinucleotide
FMN: Flavin mononucleotide
MRI: Magnetic resonance imaging
RFVT: Riboflavin transporter
SMN: Survival motor neuron
